# Red cell distribution width (RDW) is correlated to time of oxygen desaturation < 90% and length of sleep apneas in patients with sleep disorder breathing (SDB) and acute heart failure with preserved ejection fraction (HFpEF)

**DOI:** 10.3389/fcvm.2023.1045702

**Published:** 2023-02-03

**Authors:** Cosimo A. Stamerra, Emilia D'Elia, Mauro Gori, Federica Roncali, Alberto Cereda, Antonello Gavazzi, Claudio Ferri, Michele Senni

**Affiliations:** ^1^Department of Life, Health and Environmental Sciences, University of L'Aquila, L'Aquila, Italy; ^2^Cardiology Unit, Hospital Papa Giovanni XXIII, Bergamo, Italy; ^3^Fondazione Ricerca Ospedale Maggiore (FROM) Research Center, Hospital Papa Giovanni XXIII, Bergamo, Italy; ^4^Cardiology Department, University of Milano-Bicocca, Milan, Italy

**Keywords:** sleep apnea, red distribution width, oxygen saturation, acute heart failure, heart failure with preserved ejection fraction (HFpEF)

## Abstract

**Background:**

Heart failure with preserved ejection fraction (HFpEF) is very frequently associated to sleep breathing disorders (SDB). Red blood cell distribution width (RDW) has been shown to be a potential inflammatory index linked to the degree of hypoxia and oxidative stress.

**Aim:**

To identify the existence of a possible relationship between sleep apnea, oxygen saturation (SaO2) and RDW in a population of subjects affected by acute HFpEF (AHFpEF).

**Methods:**

AHFpEF patients with known history of SDB were enrolled and performed blood chemistry, echocardiography, and 24-h polysomnography (PSG).

**Results:**

A total of 34 acute HFpEF patients (mean age 72.8 +/−8.63) were enrolled in the study. A control group of 24 non-HF patients were considered. Compared to controls, HFpEF patients showed a higher mean apnea hypopnea index (AHI), with prevalence of central apneas. A moderate to severe desaturation pattern was observed in AHFpEF vs. controls. RDW was significantly higher in AHFpEF patients vs. controls (mean value 14.7 +/−2.6 % vs. 9.1 +/−2.2, *p* < 0.05). In AHFpEF, RDW showed a positive correlation with time of SaO2 < 90% (*r* = 0.35, *p* = 0.04), and with mean length of apneic events (60 +/−28 s, *r* = 0.29, *p* = 0.03).

**Conclusion:**

In patients with AHFpEF and SDB, a dependence relationship between RDW and duration of oxygen desaturation was observed, as if oxidative stress and inflammation related to RDW increase could also be linked to severity of sleep disorders in this population.

## Introduction

Anisocytosis is a condition characterized by the presence of various sizes of red blood cells (RBCs) in the peripheral blood. Red cell distribution width (RDW) provides a quantitative measure of anisocytosis, and it is associated with the presence of subclinical systemic inflammation in a variety of clinical situation such as renal impairment, nutritional deficiencies, and aging (1). Anisocytosis worsens the prognosis of cardiovascular events in patients with heart failure (HF) or in asymptomatic patients at risk of developing HF (2). Moreover, the presence of anisocytosis in heart failure with preserved ejection fraction (HFpEF), has been demonstrated to be associated with a higher frequency of non-cardiac mortality. Several HFpEF comorbidities such as renal failure, arterial hypertension, metabolic dysfunctions, neurocognitive impairment, oxidative stress, obesity, and diabetes worsen the degree of inflammation, and consequently increase RDW (3).

Sleep disordered breathing (SDB) is very common in HFpEF, with a prevalence between 50 and 70%, especially in case of obstructive sleep apneas (OSAS) (4–9). Intermittent hypoxia in OSAS triggers pro-inflammatory transcription factors, and consequent activation of inflammatory cells. To this regard, in a general population complaining snoring and witnessed apneas, it has been demonstrated a relationship between severity of OSAS and RDW that is dependent on inflammation and intermittent hypoxia, but independent from anemia (10).

Little is known regarding the role of RDW as a marker of inflammation in acute HFpEF (AHFpEF) patients with SDB, nor a potential correlation between RDW, OSAS, and oxygen saturation (SaO2) has ever been investigated in AHFpEF.

Thus, the aim of our study is to investigate a potential association between RDW, OSAS, and SaO2 in AHFpEF patients with history of SDB.

## Materials and methods

### Study population

We designed a prospective, single-center study approved by the Ethical Committee of Papa Giovanni XXIII Hospital, Bergamo, Italy. All consecutive AHFpEF patients admitted to the cardiology ward for decompensated HF between March 2019 and December 2020 were considered. Inclusion criteria were: (i) ≥18 years old; (ii) hospitalization for AHF, according to ESC guidelines (11); (iii) NYHA (New York Heart Association) class ≥ III; (iv) diuretic infusions required within 24 h from hospital admission; (v) presence of documented sleep apnea; (vi) written informed consent. Exclusion criteria were: (i) acute coronary syndrome; (ii) severe pulmonary disease; (iii) severe valvular disfunction. A control group of non HF patients with left ventricle ejection fraction (LVEF) >50% hospitalized for causes other than HF and acute myocardial ischemia was considered.

A set of biochemical tests, an echocardiogram and a 24 h polysomnography were performed in the acute phase of hospitalization, once the patient switched from intravenous to oral diuretics.

Median follow up was 18 months.

### Polysomnography, echocardiogram, and biochemical tests

Embletta diagnostic system (ResMed TM, Sidney, Australia) is a portable device providing AHI (apnea hypopnea index), AI (Apnea Index), HI (Hypopnea Index), central apnea index (CAI), obstructive apnea index (OAI) through specific sensors able to detect respiratory and abdominal movements. “Apnea” is defined as an absence of airflow for at least 10 s, “hypopnea” is defined as a 30% reduction in airflow accompanied by a decrease in SaO2 of at least 4% (12). An AHI >15 events/h was considered significant for diagnosis of SDB. For all patients, 24 h polysomnography was settled during hospitalization, at the time of switch from intravenous to oral diuretic. The variables investigated were: AHI, AI, HI, CAI (central AI), OAI (obstructive AI), oxygen desaturation index (ODI), mean and minimum SaO2, time and percentage of desaturation <90%, number of desaturation events >4 %, and minimum and maximum length of an apneic event (seconds).

All enrolled patients underwent also a transthoracic echocardiography (TTE) in the acute phase of hospitalization. All parameters of diastolic dysfunction and right ventricular (RV) function were evaluated (**Table 4**).

The biochemical tests necessary for the performance of normal clinical-care activities were detected in all patients during the acute phase of hospitalization and the follow up visits.

### Statistical analysis

PRISM 4 Software (GRAPHPAD PRISM 4.0; GraphPad Software, La Jolla, CA, USA) was used for statistical analyses of the entire population. Quantitative variables are shown by mean and standard deviation. All the variables were compared between cases and controls using non-parametric Wilcoxon tests (for continuous variables) or Fisher's exact tests (for categorical variables).

Overall, *p*-values below 0.05 were defined statistically significant.

## Results

Between March 2019 and December 2020, a total of 34 AHFpEF patients (23 men, 11 women, mean age 72.8 +/−8.63), with SDB diagnosed by an AHI > 15 events/hour in a PSG performed within the previous 3 years were enrolled in the study. They were compared to a group of 24 non-HF patients admitted to the hospital for other cardiovascular causes, with a similar LVEF. Clinical characteristics and blood chemistry values of the patients enrolled are shown in Tables 1, 2. Medical treatment previous to enrollment is described in Table 3.

**Table 1:** Clinical characteristics of the patients enrolled.

	**HFpEF (34)**		**Not-HF (24)**		***P*-value**

	***N*** **of pts**	**%**	***N*** **of pts**	**%**	
HF-“De Novo”	30	91.6	0	0.00	<0.001
Age (average, years)	72.8 +/− 8.63	/	69.3+/− 7.2	/	NS
Male	23	67	14	58.3	0.04
BMI (avarage, Kg/m^2^)	26.4+/− 3.4	/	26+/− 5.2	/	NS
Hypertension	34	100	8	33	NS
T2DM	19	58.3	2	8	0.02
Dyslipidemia	28	83.3	16	66.6	0.04
Current smoke/previous smoke	0/5	0/14	14/6	58.3/25	NS
Family history	3	8.5	10	41.6	0.04
COPD	6	16.6	0	0	NS
Atrial fibrillation	23	70	4	16.6	0.03
History of CAD	8	56.6	2	8	0.02
Charlson index (average)	5	/	2	/	NA
Lenght of hospitalization (average, days)	13.1	/	11.4	/	NA

pts, patients; HF, heart failure; BMI, body mass index; T2DM, type 2 diabetes mellitus; CAD, coronary artery disease; COPD, chronic obstructive pulmonary disease; NS, not significant; NA, not applicable.

**Table 2:** Blood chemistry values.

	**HFpEF (34)**	**Not-HF (24)**	***P*-value**
BNP admission (pg/ml)	642+/−140	91.6+/−24	<0.05
BNP discharge (pg/ml)	293+/−101	88.3+/−44	<0.05
Hb (g/L)	108.0+/−34	137.4+/−28.7	NS
RDW	14.7+/−2.6	9.1 +/−2.2	<0.05
GFR (ml/min/1.73 mq)	64.1+/−16.6	72.4+/−18.4	NS
GOT (U/L)	26.8+/−2.8	24.6+/−5.3	NS
GPT (U/L)	24.4+/−4.5	25.4+/−3.5	NS
TSH (μU/Ml)	2.2+/−3.5	1.6+/−4.2	NS
Uric acid (mg/dl)	7.6+/−0.7	5.2+/−1.2	NS
Iron (mcg/dl)	46.1+/−8.7	57.5+/−12.4	NS
Ferritin (ng/ml)	148.6+/−19.7	224.7+/−14.7	NS
Ferritin saturation (%)	17.5+/−2.7	32.1+/−6.7	NS
Total cholesterol (mg/dl)	133.1+/−76	180.6+/−92.8	NS
HDL cholesterol (mg/dl)	35.9+/−12.6	49.1+/−49.9	NS
LDL cholesterol (mg/dl)	76.6+/−34	101.3+/−47.2	NS
Tryglicerides (mg/dl)	91.4+/−42	123.7+/−32.8	<0.05
CRP (mg/dl)	1.5+/−0.9	2.2+/−1.8	NS

BNP, brain natriuretic peptide; Hb, hemoglobin; GFR, rate glomerular filtration; GOT, glutamic oxaloacetic transaminase; GPT, glutamic pyruvic transaminase; TSH, thyroid stimulating hormone; HDL, high density lipoproteins; LDL, low density lipoprotein; CRP, C-reactive protein; NS, not significant.

**Table 3:** Medical therapy.

	**HFpEF (34)**		**Not-HF (24)**		***P*-value**

	**N of pts**	**%**	**N of pts**	**%**	
ACE-I/ARB	24	70	6	25	NS
βb	31	91.6	5	20	0.03
MRA	30	90	0	0	0.001
Sacubitril/valsartan	0	0	0	0	NS
SGLT2i	0	0	0	0	NS
Statin	28	83	14	58.3	0.04
Metformin	19	58.3	2	8.3	0.03
Insulin	5	16	2	8.3	0.04
Oral Iron	13	40	0	0	0.02
Carboxymaltose ferric	19	58.3	2	8.3	0.02

ACE-I, angiotensin converting enzyme inhibitors; ARB, angiotensin receptor blocker; βB, beta-blockers; MRA, mineralocorticoid receptor antagonist; SGLT2i, sodium-glucose co-transporter-2 inhibitors.

In the AHFpEF group mean brain natriuretic peptide (BNP) at admission was 642 +/−140 ng/L. Every patient was treated with diuretic infusions and was than subjected to a 24 h PSG. In the case group vs. control group, a higher mean AHI was documented (27+/−36.7 events/hour vs. 9.8 +/−22 events/hours, *p* = 0.002), and a prevalence of central apneas was reported in AHFpEF patients (CAI = 10 +/−13.2; OAI = 3.5 +/−7.7). A moderate to severe desaturation pattern was observed in AHFpEF vs. controls (mean SaO2 = 92 +/−2.8 % vs. 93 +/−3%, *p* = 0.11; minimum SaO2 = 80.1 +/−14.2 % vs. 87.2 +/−10 %, *p* = 0.002; time SaO2 <90% = 80 +/−113 min vs. 12.2 +/−68, *p* = 0.02; Oxygen desaturation index (ODI) 22.1 +/−17.4 vs. 9.9 +/−12, *p* < 0.05). RDW was significantly higher in AHFpEF vs. non HF patients (mean value 14.7 +/- 2.6 % vs. 9.1 +/- 2.2, *p* < 0.05). In AHFpEF, RDW showed a positive correlation not only with time of SaO2 <90% (*r* = 0.35, *p* =0.04), but also with medium length of apneic events (60 +/−28 s, *r* = 0.29, *p* = 0.03). A strong positive correlation between RDW and mean pulmonary arterial pressure (PAPs 29.2 +/−12.4 mmHg, *r* = 0.54, *p* = 0.004) was observed.

In the two groups, nor C reactive protein or white blood cell count showed any significant correlation with PSG parameters related to hypoxia. Moreover, no significant correlations were documented between RDW and AHI, nor between RDW and BNP. Echocardiographic and polysomnographic values of the enrolled population are shown in Table 4. During the mean follow up of 18 months, 13 patients (38%) were admitted to hospital for worsening HF. These patients did not have a particular accentuation of basal sleep disturbances compared to those patients who were not re-admitted to the hospital.

**Table 4:** Echocardiographic and polysomnographic values.

	**HFpEF (34)**			**Not-HF (24)**			***p*** **-value for differences HFpEF vs. Not-HF**	

	**Baseline**	**Follow-up**	***p*** **-value**	**Baseline**	**Follow-up**	***p-*** **value**	**Baseline**	**Follow-up**
FE (%)	54.4	55.1	0.40	54.8	54.6	0.48	0.2	0.06
LVDV/LVSV (ml)	145/82	135/75	0.45	120/75	125/68	0.5	0.4	0.4
E/A	2.52	2.48	0.48	0.94	0.86	0.32	**0.00**	**0.01**
E/e'	15.52	13.2	0.18	10.7	10.4	0.44	**0.04**	0.06
e' septal (m/s)	0.06	0.06	0.91	0.12	0.11	0.46	**0.06**	0.09
e'lateral (m/s)	0.08	0.11	**0.01**	0.09	0.09	0.9	0.19	0.29
LAVI (ml/m^2^)	63.49	54.0	0.26	48.3	49.7	0.36	0.13	0.31
TAPSE (mm)	20.57	25.14	**0.05**	22.3	23.8	0.28	**0.03**	0.48
PAPs (mmHg)	34.57	25.02	**0.02**	17.3	17.6	0.46	**0.000**	**0.03**
AHI (events/h)	27	16.65	0.06	9.8	NE	/	**0.002**	/
CAI (events/h)	10	2.85	**0.05**	1.1	NE	/	**0.008**	/
OAI (events/h)	3.5	0.68	0.08	4.1	NE	/	0.29	/
ODI (events/h)	22.1	17.3	**0.04**	9.9	NE	/	**0.004**	/
Mean SaO2 (%)	92.0	92.8	0.21	93.0	NE	/	0.11	/
Minimum SaO2 (%)	80.1	79.6	0.39	87.2	NE	/	**0.002**	/
SaO2 <90% (min)	80	51.0	0.15	12.2	NE	/	0.02	/
Medium length of apneic event	60	50	0.2	30	NE	/	0.03	/

EF, ejection function; E, E wave; A, A wave; e', early diastolic velocity; LAVI, left atrial volume index; TAPSE, tricuspid annular plane systolic excursion; PAPs, pulmonary arterial systolic pressure; AHI, apnea hypopnea index; CAI, central apneas index; OAI, obstructive apneas index; ODI, oxygen desaturation index; SaO2, oxygen saturation; NE, not executed. Bold values denote significant *p* value.

## Discussion

In this study, we observed that AHFpEF patients seem to report a higher RDW value compared to non-HF patients with similar LVEF, and we hypothesize that RDW could be a subtle marker of inflammation in this population.

Anisocytosis, HFpEF, and SDB are three different entities, sharing many pathogenetic mechanisms.

Anisocytosis is an important predictor of morbidity and mortality in patients affected by various chronic diseases, such cardiovascular, pulmonary, and rheumatic diseases (13–16). Thus, mechanisms underlying the increase in RDW may affect outcomes in patients already affected by chronic diseases of different origins, as it happens in HFpEF. HFpEF is a complex clinical syndrome characterized by a normal LVEF in presence of a diastolic dysfunction, leading to myocardial stiffness and elevated filling pressures. Comorbidities, both cardiovascular and non-cardiovascular, are main features of HFpEF, whose phenotype directly depend on the interactions of these multiple co-existent diseases. Oxydative stress, endothelial dysfunction, and systemic inflammation are peculiar characteristics of HFpEF, in fact a chronic state of inflammation has largely been described as a substrate for the incipit, or at least the progression, of the disease (17–19).

SDB is a syndrome characterized by abnormal respiration during sleep, and it is strongly related to chronic inflammation, as intermittent hypoxia due to central or obstructive sleep apneas leads to the development of oxidative stress, endothelial dysfunction, and consequently a vicious circle enhancing both acute and chronic cardiovascular diseases (20, 21).

A connection among HFpEF, SDB, and anisocytosis could be surmised in the proinflammatory state which is typical of these pathologies. HFpEF and SDB are, in fact, characterized by hypoxia, oxidative stress, activation of inflammatory cytokines, activation of reactive oxygen species, and endothelial dysfunction (22–25), all of which lead to dyserythropoiesis with increased production of erythroblasts, with a larger size heterogeneity of RBC volumes (23).

Furthermore, subjects with HF and SDB are usually elderly and may be suffering from malnutrition, metabolic dysfunctions and chronic renal failure; these pathological conditions cause a reduced bone marrow production of RBCs, with an aggravation of anisocytosis (25–27).

In a recent study, Imai et al. evaluated the correlation between high RDW values at admission and poor outcomes due to non-cardiac events in 278 AHFpEF patients, thus acting as a long-term prognostic index (3).

In this framework, anisocytosis worsens tissue hypoxia already present both in HFpEF and in SDB, enhancing oxidative stress, apoptosis and myocardial fibrosis. Moreover, in HFpEF inflammatory molecular markers and soluble interleukin family members have been shown to be independent predictors of mortality (28–30).

### Limitations

The major limitation of this study is the small sample of the population considered, that does not allow to make substantial observations.

The absence of an HF control group is also a main limitation, but the control group we evaluated had at least a normal LVEF.

Of note, we performed a 24 h PSG, which is an added value for the registration and evaluation of the data.

## Conclusions

Our study seems to suggest that AHFpEF patients tend to have higher RDW values compared to a population of non-HF patients with similar LVEF, and that RDW could be a marker of inflammation and increase of pulmonary pressure in this population.

Additionally, our research might suggest that mechanisms underlying the increase in RDW, such as inflammation and oxidative stress, also cause desaturation in subjects affected by AHFpEF and SDB, showing the connection between RDW and both time and length of oxygen desaturation.

## Data availability statement

The raw data supporting the conclusions of this article will be made available by the authors, without undue reservation.

## Ethics statement

The studies involving human participants were reviewed and approved by Ethical Committee Hospital Papa Giovanni XXIII. The patients/participants provided their written informed consent to participate in this study.

## Author contributions

CS and ED'E: writing, editing, and conduction of the study. MG: revision. AC: statistical analysis. AG, CF, and MS: supervision. All authors contributed to the article and approved the submitted version.
